# Shaping Smiles: A Narrative Review of Crown Advancements in Pediatric Dentistry

**DOI:** 10.7759/cureus.52997

**Published:** 2024-01-26

**Authors:** Omar S Almajed

**Affiliations:** 1 Pediatric Dentistry, Imam Abdulrahman Bin Faisal University, Dammam, SAU; 2 Dental Public Health, King’s College London, London, GBR

**Keywords:** pediatric dentistry, clinical outcomes, dental materials, child psychology, oral health, bioflex crowns, resin-based composite crowns, zirconia crowns, stainless steel crowns, dental crowns

## Abstract

This review examines the evolution of dental crowns in pediatric dentistry, highlighting the transition from traditional materials such as stainless steel to aesthetic and biocompatible alternatives like zirconia, resin-based composites, and Bioflex crowns. It focuses on their importance in repairing decayed or damaged teeth and improving children's oral health and psychological well-being. The methodology involved a comprehensive literature search over the past two decades, utilizing databases including PubMed, Google Scholar, and Chat.Consensus.App, with keywords related to pediatric dental crowns.

The findings indicate that stainless steel crowns (SSCs) are valued for durability and cost-effectiveness, but they may cause hypersensitivity. Zirconia crowns are favored for biocompatibility, resistance, and aesthetics, although they are costlier and require more tooth reduction. Resin-based composite strip crowns offer a balance of aesthetics and function but have challenges in long-term stability. The review also touches on Bioflex crowns, noting their flexibility, but the limited research on their effectiveness.

In summary, the review underscores the vital role of various dental crown materials in pediatric dentistry, stressing the importance of ongoing research to enhance clinical outcomes and pediatric patient quality of life. The selection of crown materials should consider efficacy, aesthetics, and the psychosocial effects on young patients.

## Introduction and background

Dental crowns in pediatric dentistry play a crucial role not only in the restoration of decayed or damaged teeth but also in the overall oral health and psychological well-being of children. The use of dental crowns is significant in several aspects, including identification in forensic pediatric dentistry, age estimation, and the analysis of dental evidence [1].

Oral rehabilitation using dental crowns, such as stainless steel crowns (SSCs) and resin-filled celluloid forms, has been shown to improve psychological behavior, reduce to[oth sensitivity, and enhance eating habits in children with conditions like amelogenesis imperfecta [2]. The evolution of dental crowns has led to the introduction of materials like zirconia, which combine biocompatibility, resistance, and ideal aesthetic outcomes, especially important for the rehabilitation of primary teeth with significant structural loss [3].

The importance of dental crowns in pediatric dentistry extends beyond mere restoration. They are integral to managing decay, particularly with innovative approaches like Hall crowns, which have been advocated for their superiority in managing decay in primary teeth compared to conventional restorations [4]. Despite the clear benefits, there is evidence that general dentists' knowledge about the indications for SSCs is sufficient, but their actual use in practice is low, indicating a gap between knowledge and application [5].

This review aims to examine the advancements in dental crown materials within pediatric dentistry, tracing the evolution from traditional stainless steel to the introduction of more aesthetic and biocompatible options like zirconia, resin-based composites, and the innovative Bioflex crowns. It will focus on assessing how these material advancements have influenced treatment outcomes, patient satisfaction, and the overall impact on the oral health and well-being of young patients.

## Review

Materials and methods

This review on pediatric dental crowns involved a comprehensive literature search across databases, including PubMed, Google Scholar, and the Chat.Consensus.App plugin. The search strategy focused on identifying peer-reviewed articles, clinical trials, and retrospective studies published within the last two decades. Key search terms included "pediatric dental crowns," "stainless steel crowns," and "zirconia crowns in children," among others.

The inclusion criteria were stringently defined to ensure relevance and focus. Selected articles were required to be published in English, concentrate on pediatric patients aged 0-12 years, and compare different materials used for dental crowns in children. Essential metrics for inclusion encompassed data on longevity, effectiveness, and patient outcomes related to these crowns. Studies were excluded if they did not specifically address pediatric dental crowns, were older than 20 years (except when providing historical context), or were non-peer-reviewed literature.

Data extraction and analysis involved collating information on the types of dental crown materials, clinical indications, comparative longevity and effectiveness, patient satisfaction, and aesthetic outcomes. These data were systematically categorized based on material type and clinical outcomes. A qualitative synthesis approach was employed to compare and contrast findings from various studies, with a focus on the evolution of materials used in pediatric dental crowns, their clinical performance, and their impact on patient satisfaction and dental health outcomes.

Instead of a detailed quality assessment, studies were selected based on their relevance, contribution to the field, and overall robustness. While a formal quality appraisal was not conducted, the general quality and scientific rigor of the studies were considered in the synthesis and discussion of the review, ensuring a balanced and comprehensive overview of the topic.

Review

Stainless Steel Crowns

SSCs are a fundamental component in pediatric dentistry, primarily due to their durability and cost-effectiveness [6]. They offer full crown coverage, significantly enhancing caries prevention by protecting the entire tooth, and are more durable than multisurface amalgam restorations [6]. SSCs are particularly useful in restoring pulp-treated teeth, reducing the risk of tooth fracture due to weakened dentin [6]. They are also preferred in dental treatments under general anesthesia, as they allow for more efficient treatment and shorter operation times, which is beneficial for child cooperation [6]. However, SSCs are not without their drawbacks. One notable issue is the potential for microleakage in the marginal area due to their prefabricated nature, although this is generally not considered critical compared to their advantages [6].

In terms of clinical outcomes, SSCs have been compared favorably to other options like zirconia crowns, especially in terms of cost-effectiveness and ease of placement [7]. However, they do have aesthetic limitations, which can be a concern for parents [7]. To address this, pre-veneered SSCs with a composite-bonded veneer are available, offering a more pleasant appearance, albeit at a higher cost and requiring more preparation [6]. The key characteristics of SSCs are summarized in Table 1.

**Table 1:** Characteristics of Stainless Steel Crowns (SSCs) in Pediatric Dentistry

Feature	Description
Durability	High
Cost-effectiveness	High
Full crown coverage	Yes
Caries prevention	Excellent
Aesthetic	Limited
Risk of hypersensitivity (nickel)	Yes
Postoperative discomfort	Moderate
Indications	Pulp-treated teeth, children with dental defects, etc.
Complications	Potential for microleakage, gingival inflammation

One significant concern with SSCs is the risk of delayed hypersensitivity reactions, particularly due to nickel content, which can cause perioral skin eruptions [3]. These typically heal within a week after crown removal [8]. Additionally, SSCs have been associated with moderate postoperative discomfort, which can be managed with painkillers [6].

Despite these concerns, SSCs are still widely used and are considered a successful treatment modality in pediatric dentistry [9]. They are particularly indicated in children with conditions like hypophosphatemia, heritable dental defects (e.g., amelogenesis imperfecta, dentinogenesis imperfecta), and enamel hypoplasia [6]. The use of SSCs should be carefully considered, especially in cases where gingival inflammation is a potential complication [10]. Proper adaptation of crown margins can minimize irritation and periodontal problems [10].

In summary, SSCs remain a fundamental choice in pediatric dentistry due to their durability and cost-effectiveness. While they offer reliable full crown coverage and are effective in caries prevention, their aesthetic limitations and potential for hypersensitivity are notable drawbacks [9]. Their use is particularly advantageous in specific clinical scenarios, such as in pulp-treated teeth and children with dental defects [9].

Zirconia Crowns

Zirconia crowns have become increasingly popular in pediatric dentistry, known for their biocompatibility, resistance, and excellent aesthetic outcomes [11]. They provide good middle-term success and enhanced patient satisfaction up to three years post-insertion, offering an aesthetically pleasing alternative to conventional metal-ceramic crowns [12]. Parents report high satisfaction with zirconia crowns due to their improved gingival and periodontal health, retention, fracture resistance, and color stability [13]. Additionally, zirconia crowns are more gingival-friendly and aesthetically appealing than stainless steel crowns, making them a preferred choice for posterior full coronal restoration [14]. However, they come with higher costs and require more tooth reduction compared to other options [7]. The detailed features of zirconia crowns are presented in Table 2.

**Table 2:** Characteristics of Zirconia Crowns in Pediatric Dentistry

Feature	Description
Aesthetic outcome	Excellent
Biocompatibility	High
Cost	Higher than SSCs
Tooth reduction	More than SSCs
Gingival health	Better compared to SSCs
Parental satisfaction	High
Resistance to fracture	High
Plaque accumulation	Less compared to SSCs
Clinical indications	Early childhood caries, traumatic dental injuries, etc.

Zirconia crowns in primary teeth have shown superior mechanical properties, such as durability and resistance to fracture and wear, in addition to providing a natural aesthetic to the rehabilitated tooth [15]. They are indicated for primary teeth affected by early childhood caries, traumatic dental injuries, and developmental defects of enamel [16]. These crowns present resistance, durability, higher aesthetic properties, good gingival health, and biocompatibility, along with good parental and children's acceptance and satisfaction [17]. However, some limitations are mentioned, including higher costs, the necessity for greater amounts of tooth reduction, and the impossibility of adjustments [3].

Clinical evaluations have shown that zirconia crowns are comparable to preformed stainless steel crowns (SSCs) in primary molars, with better gingival scores and excellent color match [18]. They have been found to prevent adhesion of *Streptococcus mutans* onto their surface, reducing plaque accumulation around the crown and inflammation of surrounding gingiva compared to conventional stainless steel crowns [19]. This makes zirconia crowns a viable option for reducing the overall microbial density and prevalence in the oral cavity, thus reducing the caries risk in the long term [20].

To sum up, zirconia crowns are a promising alternative to other restorative materials and crowns in pediatric dentistry. They have shown higher properties and performance in different clinical aspects and great parental satisfaction [21]. However, the choice between zirconia and other types of crowns should be based on a comprehensive understanding of their benefits and limitations, ensuring the best possible outcome for pediatric dental patients [22].

Resin-Based Composite Strip Crowns

Resin-based composite strip crowns offer a balance of aesthetics and function, enhancing the quality of life for pediatric patients and their families [23]. They are cost-effective, easily made, and quickly restore aesthetics and function [23]. However, their long-term stability can be challenging, requiring careful consideration in their application [24]. The main attributes and considerations for resin-based composite strip crowns are outlined in Table 3.

**Table 3:** Characteristics of Resin-Based Composite Strip Crowns in Pediatric Dentistry

Feature	Description
Aesthetic outcome	Excellent
Cost-effectiveness	High
Long-term stability	Challenging
Clinical time	Shorter
Parental satisfaction	Generally high
Main disadvantages	Color stability, durability
Indications	Primary anterior teeth with extensive coronal destruction
Restoration success rate	High (over 80% at final follow-up)

These crowns are a viable alternative for restoring primary anterior teeth with extensive coronal destruction, presenting advantages like satisfactory aesthetic results, being quickly and easily made, without a laboratorial phase, and not being very expensive [25]. They can be effectively utilized for various restorations, including preventive resin restorations, moderate Class II restorations, Class III restorations, Class IV restorations, Class V restorations, and strip crowns [26]. Tooth isolation to prevent contamination is a critical factor, and high-risk children may not be ideal candidates for resin-based composite restorations [27].

The preparation of composite resin crowns with the help of strip crowns results in shorter clinical time, better aesthetic results, and the restoration of the patient’s smile and self-esteem [27]. More than 80% of these restorations were judged to be successful at the final follow-up examination [28]. The technique offers the advantages of using one restorative material, improving aesthetics, and reducing chair time and costs [29].

Parental satisfaction with bonded resin composite strip crowns for primary incisors is generally excellent [30]. However, satisfaction with regard to color received the lowest rating [31]. The complete loss of strip crowns is mainly related to eating bites [32]. The bonded resin composite strip crown is the most aesthetic of all the restorations available for the treatment of severely decayed primary incisors [33].

Resin-based composite strip crowns strike a balance between aesthetics and functionality, offering a cost-effective solution for restoring primary anterior teeth. While they excel in aesthetic outcomes and are generally well-received by parents, challenges in long-term stability and color stability are concerns that need consideration in their application [34,35].

Bioflex Crowns

Bioflex crowns, a newer development in pediatric dentistry, are known for their flexibility and adaptability, combining the properties of stainless steel and zirconia crowns [35]. These crowns are based on a biocompatible hybrid resin polymer, which addresses concerns related to ductility, color stability, and durability that are often associated with fiberglass-reinforced composite crowns. Notably, Bioflex crowns offer a "flex fit" adaptation over the anatomic cervical convexity of primary teeth, similar to stainless steel crowns, but with the added benefit of a more esthetic appearance and conservative tooth preparation, comparable to pediatric zirconia crowns. However, there is currently a lack of comprehensive studies assessing the properties of Bioflex crowns, their impact on clinical outcomes, and parental satisfaction. This gap underscores the need for focused research to evaluate how they compare with traditional options like stainless steel and zirconia crowns, especially considering their potential advantages in esthetics and tooth conservation. Preliminary case reports suggest promising results in terms of ease of placement, esthetic appeal, and clinical performance, but more extensive research is needed to fully establish their efficacy and long-term outcomes in pediatric dental care [36].

Bioflex crowns represent an innovative development in pediatric dentistry, combining the flexibility and adaptability of both stainless steel and zirconia crowns. While they show promise in terms of esthetics and conservative tooth preparation, the limited research on their long-term effectiveness and clinical outcomes suggests a need for further studies to fully establish their role in pediatric dental care.

Furthermore, when considering the broader range of options in pediatric dental restorations, it is evident that each type of crown (stainless steel crowns (SSCs), zirconia crowns, and resin-based composite strip crowns) comes with its own set of unique features, advantages, and disadvantages. The choice of the most appropriate crown type is influenced by a variety of factors, including aesthetic requirements, durability, cost considerations, and specific clinical indications. To facilitate this crucial decision-making process, Table 4 provides a comprehensive comparison of these crown types. This table succinctly summarizes their key characteristics, offering a quick and effective overview for dental professionals (see Table 4). This comparative analysis is designed to assist in selecting the most suitable crown type for pediatric patients, ensuring the best possible outcome tailored to the individual needs and circumstances of each patient.

**Table 4:** Comparison of Crown Types in Pediatric Dentistry

Feature	Stainless Steel Crowns (SSCs)	Zirconia Crowns	Resin-Based Composite Strip Crowns
Aesthetic appeal	Limited	Excellent	Excellent
Durability	High	High	Moderate
Cost-effectiveness	High	Moderate (higher than SSCs)	High
Caries prevention	Excellent	Good	Good
Risk of hypersensitivity	Yes (nickel content)	No	No
Postoperative discomfort	Moderate	Low	Low
Gingival health	Moderate	Better than SSCs	Good
Clinical indications	Pulp-treated teeth, children with dental defects	Early childhood caries, traumatic dental injuries	Primary anterior teeth with extensive coronal destruction
Tooth reduction required	Minimal	More than SSCs	Minimal
Parental satisfaction	Moderate	High	High
Long-term stability	High	High	Challenging
Plaque accumulation	Moderate	Less compared to SSCs	Moderate
Special considerations	Potential for microleakage; aesthetic concerns	Higher cost; requires more tooth reduction	Color stability; durability concerns

## Conclusions

In conclusion, dental crowns play a vital role in pediatric dentistry, not just in restoring damaged teeth but also in supporting the overall oral health and psychological well-being of children. From traditional stainless steel crowns to modern zirconia and resin-based composites, each type offers unique benefits in terms of aesthetics, durability, and biocompatibility. The recent introduction of Bioflex crowns, combining features of stainless steel and zirconia, highlights ongoing advancements, although further research is needed to fully understand their potential. The choice of crown type should be guided by factors such as aesthetic needs, durability, cost, and clinical indications. This review underscores the importance of selecting the most appropriate crown type for each pediatric patient, aiming to achieve the best possible treatment outcomes and quality of life.
